# New Books

**Published:** 2010-07

**Adapting to the Impacts of Climate Change**

America’s Climate Choices: Panel on Adapting to the Impacts of Climate Change; National Research Council

Washington, DC:National Academies Press, 2010. 325 pp. ISBN: 978-0-309-14591-6, $56.95

**Advancing the Science of Climate Change**

America’s Climate Choices: Panel on Advancing the Science of Climate Change; National Research Council

Washington, DC:National Academies Press, 2010. 506 pp. ISBN: 978-0-309-14588-6, $56.95

**Air**

John Knechtel

Cambridge, MA:MIT Press, 2010. 320 pp. ISBN: 978-0-262-01466-3, $15.95

**Bottled and Sold: The Story Behind Our Obsession with Bottled Water**

Peter Gleick

Washington, DC:Island Press, 2010. 288 pp. ISBN: 978-1-59726-528-7, $26.96

**Bridging the Evidence Gap in Obesity Prevention: A Framework to Inform Decision Making**

Shiriki K. Kumanyika, Lynn Parker, Leslie J. Sim, eds.

Washington, DC:National Academies Press, 2010. 340 pp. ISBN: 978-0-309-14989-1, $55

**Design for Ecological Democracy**

Randolph T. Hester

Cambridge, MA:MIT Press, 2010. 528 pp. ISBN: 978-0-262-51500-9, $21.95

**Disrupted Networks: From Physics to Climate Change**

Bruce J West, Nicola Scafetta

Hackensack, NJ:World Scientific, 2010. 316 pp. ISBN: 978-981-4304-30-6, $68

**Handbook of Renewable Energy Technology**

Ahmed F. Zobaa, Rarnesh Bansal

Hackensack, NJ:World Scientific, 2010. 700 pp. ISBN: 978-981-4289-06-1, $185

**Health in the European Union: Trends and Analysis**

P. Mladovsky, S. Allin, C. Masseria, C. Hernández-Quevedo, D. McDaid, E. Mossialos

Geneva:WHO Press, 2010. 189 pp. ISBN: 978-9-2890-4190-4, $50

**Ignorance and Surprise: Science, Society, and Ecological Design**

Matthias Gross

Cambridge, MA:MIT Press, 2010. 256 pp. ISBN: 978-0-262-01348-2, $30

**Indoor Air Pollution**

Robert Maynard, Paul Harrison, Isabella Myers

Hackensack, NJ:World Scientific, 2010. 400 pp. ISBN: 978-1-84816-493-2, $ 115

**Limiting the Magnitude of Future Climate Change**

America’s Climate Choices: Panel on Limiting the Magnitude of Future Climate Change; National Research Council

Washington, DC:National Academies Press, 2010. 258 pp. ISBN: 978-0-309-14597-8, $56.95

**Preparing for Climate Change**

Michael D. Mastrandrea, Stephen H. Schneider

Cambridge, MA:MIT Press, 2010. 96 pp. ISBN: 978-0-262-01488-5, $14.95

**Research Tools in Natural Resource and Environmental Economics**

Amitrajeet A. Batabyal, Peter Nijkamp, eds.

Hackensack, NJ:World Scientific, 2010. 400 pp. ISBN: 978-981-4289-22-1, $110

**Review of the Department of Defense Enhanced Particulate Matter Surveillance Program Report**

Committee for Review of the DOD s Enhanced Particulate Matter Surveillance Program Report; National Research Council

Washington, DC:National Academies Press, 2010. 200 pp. ISBN: 978-0-309-15413-0, $44.50

**Towards a Livable and Sustainable Urban Environment: Eco-Cities in East Asia**

Liang Fook Lye, Gang Chen

Hackensack, NJ:World Scientific, 2010. 200 pp. ISBN: 978-981-4287-76-0, $58

